# Efficacy of epetraborole against *Mycobacteroides abscessus* in a mouse model of lung infection

**DOI:** 10.1128/aac.00648-24

**Published:** 2024-07-17

**Authors:** Binayak Rimal, Christopher K. Lippincott, Chandra M. Panthi, Yi Xie, Tiffany R. Keepers, MRK Alley, Gyanu Lamichhane

**Affiliations:** 1 Division of Infectious Diseases, Department of Medicine, School of Medicine, Johns Hopkins University, Baltimore, Maryland, USA; 2 Center for Nontuberculous Mycobacteria and Bronchiectasis, School of Medicine, Johns Hopkins University, Baltimore, Maryland, USA; 3 AN2 Therapeutics, Inc., Menlo Park, California, USA; St. George's, University of London, London, United Kingdom

**Keywords:** *Mycobacterium abscessus*, epetraborole

## Abstract

*Mycobacteroides abscessus* (*Mab* or *Mycobacterium abscessus*) is a fast-growing mycobacterium that is ubiquitous in the environment and can cause opportunistic disease in people with lung comorbidity and immunodeficiency. There are no Food and Drug Administration-approved drugs for this disease, and repurposed antibiotics have a poor microbiological response. To address the need for effective new antibiotics, we determined the efficacy of epetraborole (EBO) against three *Mab* clinical isolates in a mouse model of lung *Mab* infection. Reduction in lung *Mab* burden over 4 weeks of treatment was the study end point. EBO was administered orally once daily at doses of 25 and 50 mg/kg, which achieved exposures approximating the once-daily dosing of 250 mg and 500 mg, respectively, in humans. EBO administration led to a gradual reduction in the lung *Mab* burden. After 4 weeks of treatment, the efficacies of 25 and 50 mg/kg EBO against isolates ATCC 19977 and M9501 were comparable. However, against isolate M9530, 50 mg/kg EBO was more efficacious than 25 mg/kg and comparable with parenteral imipenem, one of the most efficacious antibiotics against *Mab*. We also undertook a dose-ranging study by evaluating the efficacies of once-daily oral administration of 0.5, 5, 10, 25, and 100 mg/kg EBO against M9501 over 4 weeks. Once-daily oral 100 mg/kg EBO was as effective as twice-daily 100 mg/kg imipenem injection. Our study suggests that EBO could address the unmet need for effective oral treatment options for *Mab* lung disease, given the high rates of *Mab* drug resistance and limited tolerable intravenous options.

## INTRODUCTION

*Mycobacteroides abscessus* (*Mab* also known as *Mycobacterium abscessus*) is a rapidly growing non-tuberculous mycobacterium complex comprising three subspecies (*abscessus, massiliense,* and *bolletii*) that causes opportunistic lung and soft-tissue infections (1). Individuals with underlying lung conditions such as cystic fibrosis, bronchiectasis, and chronic obstructive pulmonary disease, as well as immunodeficiency, are at a higher risk of developing *Mab* lung disease. Incidence of *Mab* lung disease is rising in the US, and among non-tuberculous mycobacteria, *Mab* is the second most commonly identified species (2). There are currently no Food and Drug Administration-approved treatments for *Mab* disease. The recommended treatments involve multi-drug combinations administered in phases over months to years (3, 4). However, these treatments come with challenges, including the need for daily intravenous administration in the initial phase, significant adverse effects, the development of inducible resistance to key drugs, and logistical difficulties in administering drugs over extended periods (5, 6). Thus, there is a pressing need to develop new and more effective drugs to address this critical healthcare challenge.

Epetraborole (EBO) is a benzoxaborole, a class of compounds also known as OBORT inhibitors (7) in that they inhibit leucyl-tRNA synthetase (LeuRS) function. Benzoxaboroles have been shown to inhibit LeuRS in *Mycobacterium tuberculosis* (8)*,* Gram-negative bacteria (9), and fungi (7). LeuRS and its inhibitors present an unexplored opportunity to develop a new treatment against *Mab* disease. *In vitro* activity of EBO against *Mab* was identified in studies that screened a drug library known as the “Pandemic Response Box” (10–12). Co-crystal structure of EBO and *Mab* LeuRS shows EBO bound to the editing active site in LeuRS (11) similar to what has been observed with LeuRS from *M. tuberculosis* (8) and *Escherichia coli* (9). Two independent studies identified missense mutations in the LeuRS editing domain as the basis for high-level resistance to EBO (11, 13). Initial proof-of-concept assessment of the efficacy of EBO was undertaken in an acute SCID mouse model of *Mab* infection in two independent studies (11, 13). In one study, once-daily oral administration of 10 mg/kg for 10 days produced <1 log_10_ CFU reduction in the lungs of mice (11). In another study, two different doses of EBO were administered once daily by oral gavage for 10 days. After 10 days of treatment, 150 mg/kg and 300 mg/kg EBO reduced lung *Mab* burden by ~0.3 and ~0.7 log_10_ CFU, respectively (13). In these studies, EBO administration was initiated 1 day following intranasal inoculation of *Mab,* which represents an acute infection.

The objective of this study was to assess the efficacy of EBO in a mouse model that more closely mimics *Mab* lung disease and its treatment in humans. We have used a genetically immunocompetent mouse, C3HeB/FeJ, that is mildly immunosuppressed with corticosteroid to permit *Mab* proliferation in the lungs but imposes sufficient immune response to develop tissue pathology at chronic stages of disease as is seen in humans (14). To replicate the natural infection route in humans, mice were exposed to *Mab* aerosol for infection, and antibiotic treatment was initiated after 1 week of infection progression. This mouse model reproduces the efficacy of the standard-of-care antibiotics and has been used for evaluating the efficacy of experimental agents (15–19).

## RESULTS

### MICs of EBO against *M. abscessus* clinical isolates

We determined the minimum inhibitory concentration (MIC) of EBO against nine *Mab* isolates using the Clinical and Laboratory Standards Institute (CLSI) guidelines (20). Eight *Mab* isolates recovered from bronchiectasis or cystic fibrosis patients between 2005 and 2015 at the Johns Hopkins University Hospital were included. In addition, *Mab* strain ATCC 19977 was included as it is frequently considered in laboratory studies as a reference for *Mab* (21). The nine *Mab* isolates were subspeciated using whole genome sequencing: six belong to *abscessus* subspecies and three belong to *massiliense* subspecies (22). Mean MIC was calculated from data from two biological and two technical replicates (Table 1; Table S1). The MIC of EBO against *M. peregrinum* ATCC 700686 was 0.125 µg/mL. The MIC of EBO against seven of the nine *Mab* isolates was in the same range, 0.031–0.0625 µg/mL, as reported against other *Mab* isolates (12). However, against two isolates, M9529 and M9507, EBO’s MIC was 1.0 µg/mL and >2 µg/mL, respectively.

**TABLE 1:** MIC of epetraborole (EBO) against *M. abscessus* and *M. peregrinum* isolates^*a*^

Isolate ID	Isolate	EBO average MIC (µg/mL)
ATCC 19977	*Mycobacterium abscesssus* subspecies *abscessus*	0.0625
M9501	*Mycobacterium abscesssus* subspecies *abscessus*	0.0625
M9503	*Mycobacterium abscesssus* subspecies *abscessus*	0.0625
M9507	*Mycobacterium abscesssus* subspecies *abscessus*	>2
M9529	*Mycobacterium abscesssus* subspecies *abscessus*	1
M9530	*Mycobacterium abscesssus* subspecies *abscessus*	0.0625
M9502	*Mycobacterium abscesssus* subspecies *massiliense*	0.0625
M9509	*Mycobacterium abscesssus* subspecies *massiliense*	0.0313
M9514	*Mycobacterium abscesssus* subspecies *massiliense*	0.0625
ATCC 700686	*Mycobacterium peregrinum*	0.125

^*a*^ MIC of EBO was determined against six *M. abscessus* subspecies *abscessus,* and three *M. abscessus* subspecies *massiliense. Mycobacterium peregrinum* was included as an internal control comparator. MIC was determined in accordance with the Clinical and Laboratory Standard Institute guidelines. Average MIC was calculated from four MICs using two biological repeats and two technical repeats included in the biological repeats.

Amino acid substitutions in LeuRS have been shown to confer EBO resistance in *Mab* (11, 13). Since the EBO MICs for M9529 and M9507 are significantly higher than for ATCC 19977, we compared the LeuS sequences in these three isolates. M9529 and M9507 contain two amino acid substitutions, A315V and T355A (Fig. S1), which localize in the LeuRS editing domain, but neither substitutions were reported in the prior reports (11, 13). In addition to these mutations, there were numerous single nucleotide polymorphisms, insertions, and deletions scattered throughout the genomes of the clinical isolates. As a result, we were unable to attribute any specific mutation(s) in the genomes of M9529 and M9507 to the increased EBO MIC observed in these isolates.

### Efficacy of EBO in a mouse model of lung *M. abscessus* infection

The efficacy of EBO was evaluated against three *Mab* isolates, subspecies *abscessus*, ATCC 19977, M9501, and M9530 in a mouse model of lung *Mab* infection (14). Mean *Mab* burden of each isolate in mouse lungs in each treatment group and statistical assessment of mean *Mab* burden between each treatment group at each time point are shown in Tables S2 and S3, respectively. ATCC 19977 was included in the efficacy assessment as it is the first *Mab* isolate archived at the ATCC and has served as the default laboratory reference strain for *Mab* (21). As ATCC 19977 was isolated from a knee abscess, it is debatable how closely this isolate represents *Mab* seen in the clinic today in patients with lung disease. M9501 and M9530 were isolated between 2005 and 2015 from patients with lung disease. M9501 and M9530 were included to determine the efficacy of EBO against isolates that exhibit varying MICs against some of the most frequently used standard-of-care drugs such as clarithromycin, azithromycin, and imipenem (3, 4). The MICs of clarithromycin, azithromycin, and imipenem against M9501 are <0.06 µg/mL, 0.5 µg/mL, and 16 µg/mL, against M9530 are 3 µg/mL, 64 µg/mL, and 48 µg/mL, and against ATCC 19977 are 1 µg/mL, 16 µg/mL, and 24 µg/mL, respectively (17). The MICs of other standard-of-care drugs such as amikacin, clofazimine, and linezolid against the three isolates were similar. These isolates have been used in studies to assess the efficacies of experimental drugs and therefore provide a basis to compare findings from different studies (17, 18). Reduction in *Mab* burdens in the lungs of mice at the conclusion of 1, 2, and 4 weeks of treatment was considered end points.

**Fig 1:**
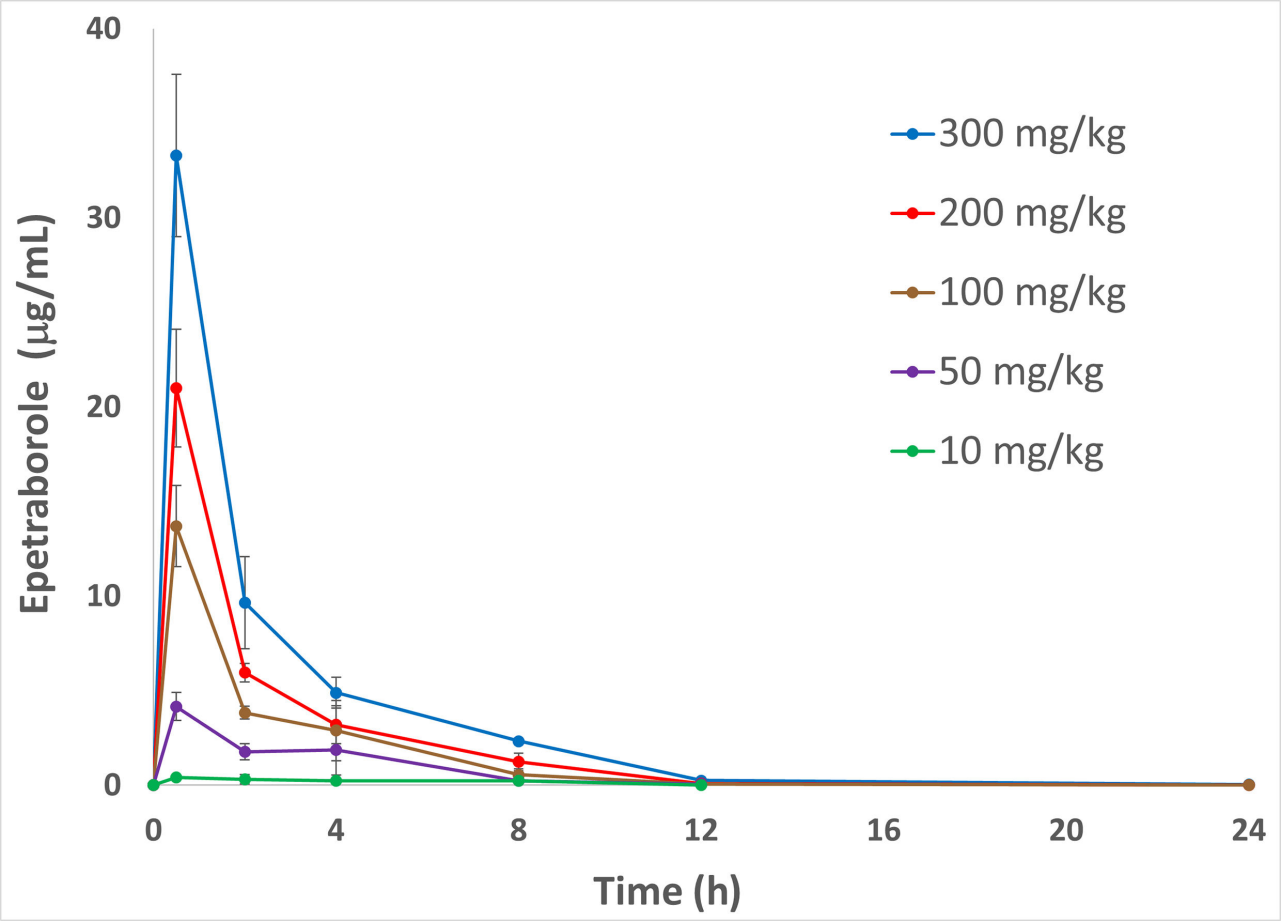
Plasma concentrations of epetraborole (mean ± standard deviation) versus plasma sampling time points following administration of Epetraborole, 10–300 mg/kg bolus, via oral gavage in C3HeB/FeJ mice are shown.

EBO is under evaluation for efficacy and safety in a Phase 2/3 clinical study for the treatment of treatment-refractory *Mycobacterium avium* complex lung disease (MAC-LD) at a dose of 500 mg orally once daily (NCT05327803). To evaluate a clinically relevant dose of EBO in the *Mab* mouse model, the pharmacokinetics (PK) of EBO in C3HeB/FeJ mice were determined so that a dose for the mouse could be chosen that would result in plasma AUC exposures similar to the exposures observed with 500 mg oral dose in humans (Fig. 1; Table 2) (23). PK studies performed in satellite animals with doses from 10 to 300 mg/kg showed that the EBO exposures achieved in this mouse strain were linear, and doses of 50 mg/kg achieved exposures that would be equivalent to the 500 mg dose in humans. The 25 mg/kg dose was chosen because the calculated exposure would be equivalent to that in humans at the 250 mg dose, which is the current size of the EBO tablets being used in the MAC-LD clinical trial.

**TABLE 2:** PK parameters in C3HeB/FeJ mice of epetraborole administered via oral gavage

Epetraborole dose (mg/kg)	C_max_ (μg/mL)^*a*^	T_max_ (h)^*b*^	T_1/2_ (h)^*c*^	AUC_last_ (h*μg/mL)^*d*^
10	0.42	0.5	nd^*e*^	2.70
50	4.16	0.5	1.92	13.8
100	13.7	0.5	1.55	31.7
200	21.0	0.5	1.70	46.7
300	33.3	0.5	1.99	76.4

^*a*^ C_max_: maximum concentration of epetraborole in plasma.

^*b*^ T_max_: time required for epetraborole concentration to reach its maximum.

^*c*^ T_1/2_: half-life of epetraborole in plasma.

^*d*^ AUC_last_: Area under the curve up to the last time point.

^*e*^ nd: not determined.

### Efficacy against *Mab* ATCC 19977

**Fig 2:**
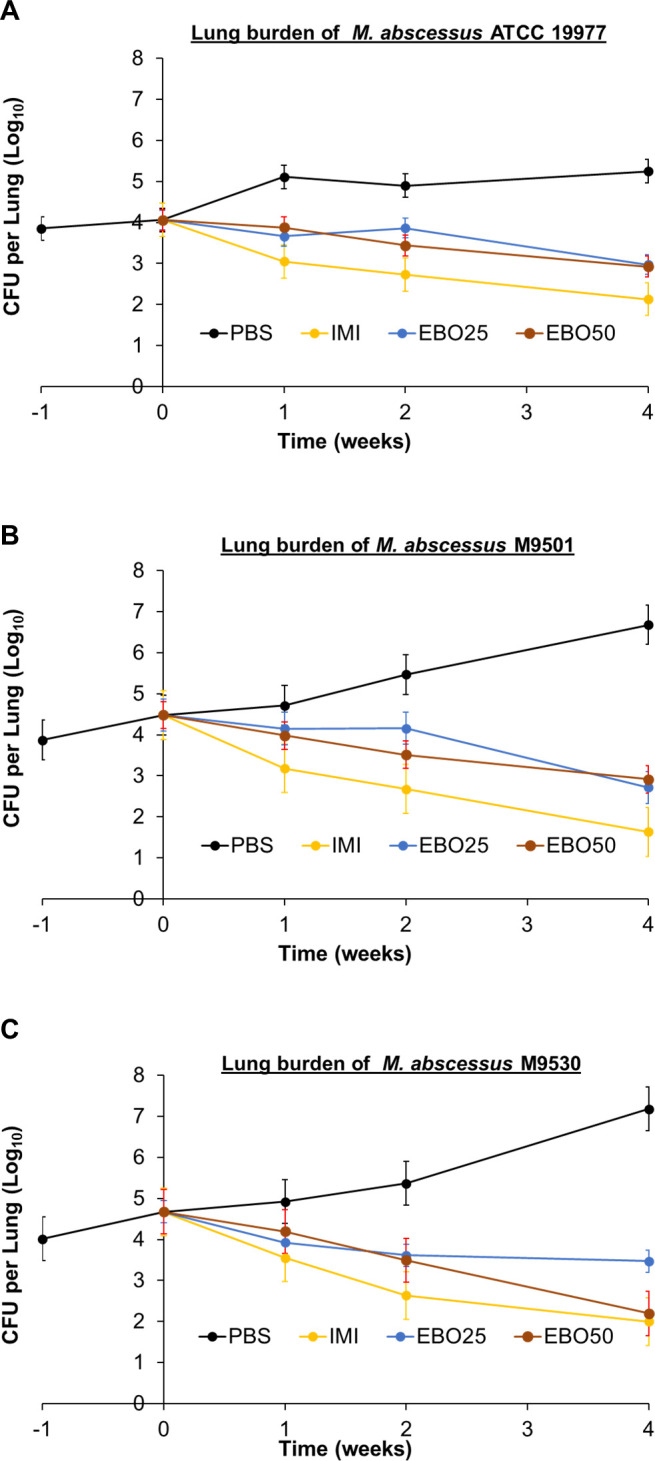
*M. abscessus* burden in the lungs of mice infected with isolates ATCC 19977 (**A**), M9501 (**B**), and M9530 (**C**). Time point week −1 represents 24 h after infection with *M. abscessus* via the aerosol route. Time point week 0 represents 1 week after infection and the day of treatment initiation. Time points weeks 1, 2, and 4 represent the end of 1, 2, and 4 weeks of treatment with once-daily 1× PBS (PBS), twice-daily 100 mg/kg subcutaneous imipenem (IMI), once-daily 25 mg/kg oral EBO (EBO25), or once-daily 50 mg/kg oral EBO (EBO50). Mean *Mab* burden in the lungs and standard error are shown (per group per time point, *n* = 5 at weeks −1, 0, + 1, and +2, and *n* = 10 at week +4).

In mice treated with PBS, lung burden of ATCC 19977 increased gradually throughout with a net increase of 1.4 log_10_ CFU at the end of the study duration (Fig. 2A). In mice that received imipenem, *Mab* lung burden decreased throughout the treatment period and resulted in a 1.9 log_10_ CFU reduction at the completion of 4 weeks of treatment. Both 25 mg/kg and 50 mg/kg EBO produced a net reduction of 1.1 log_10_ CFU at the end of 4 weeks. Therefore, 25 mg/kg and 50 mg/kg EBO exhibited comparable efficacy against ATCC 19977.

### Efficacy against *Mab* M9501

In mice treated with PBS, lung burden of M9501 increased gradually throughout with a net increase of 2.2 log_10_ CFU at the end of the study duration (Fig. 2B). Imipenem reduced M9501 lung burden by 2.9 log_10_ CFU at the end of 4 weeks of treatment. While 50 mg/kg EBO produced a larger reduction in lung CFU burden at the end of 2 weeks of treatment, 25 mg/kg and 50 mg/kg EBO produced statistically similar (*P* = 0.3) net reduction of 1.8 log_10_ and 1.6 log_10_ CFU, respectively, at the end of 4 weeks of treatment. Therefore, 25 mg/kg and 50 mg/kg EBO exhibited comparable efficacy against M9501.

### Efficacy against *Mab* M9530

In mice treated with PBS, lung burden of M9530 increased gradually throughout with a net increase of 2.5 log_10_ CFU at the end of study duration (Fig. 2C). Although the MIC of imipenem against M9530 is 48 µg/mL and would be classified as resistant according to the CLSI guidelines (20), imipenem was bactericidal against this isolate and reduced its lung burden by 2.7 log_10_ CFU at the end of the study. Similar efficacy of imipenem against M9530 was reported in a prior study (17). While 25 mg/kg and 50 mg/kg doses of EBO exhibited similar bactericidal efficacy against M9530 during the first 2 weeks, at the end of 4 weeks of treatment, 25 mg/kg and 50 mg/kg doses of EBO reduced M9530 lung burden by 1.2 log_10_ and 2.5 log_10_ CFU, respectively, compared with the lung burden at the time of treatment initiation. Against M9530, the efficacy of 50 mg/kg EBO administered orally once daily was statistically similar (*P* = 0.18) to the efficacy of twice-daily injection of 100 mg/kg imipenem, a drug that is highly bactericidal and a frontline standard-of-care treatment of *Mab* lung disease.

### Efficacy of 0.5, 5, 10, 25, and 100 mg/kg EBO

**Fig 3:**
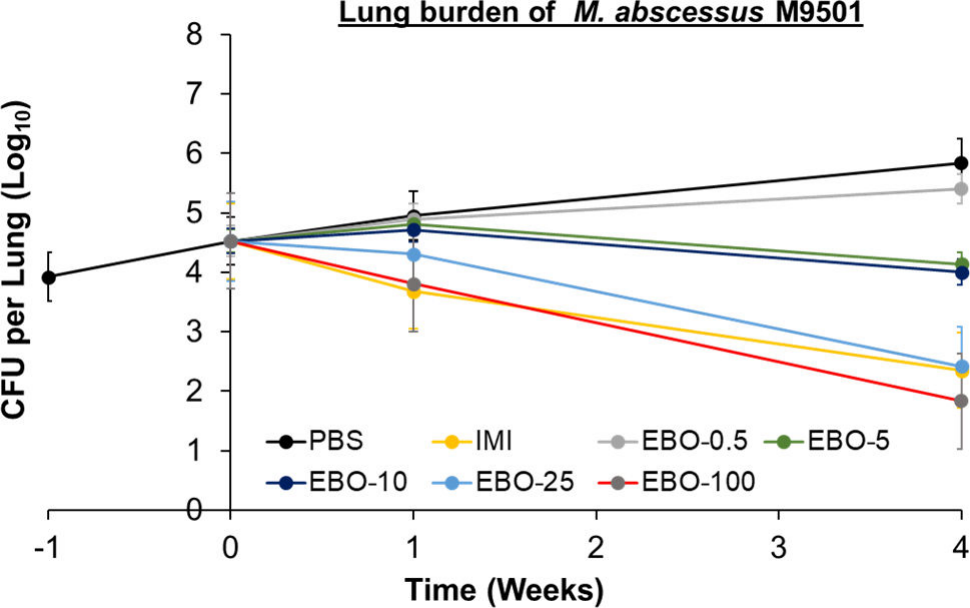
*M. abscessus* burden in the lungs of mice infected with isolate M9501. Time point week −1 represents 24 h after infection. Time point week 0 represents 1 week after infection and the day on which treatments were initiated. Time points weeks 1 and 4 represent the end of 1 and 4 weeks of treatment with once-daily 1× PBS (PBS), twice-daily 100 mg/kg intravenous imipenem (IMI), once-daily oral EBO at doses 0.5 mg/kg (EBO-0.5), 5 mg/kg (EBO-5), 10 mg/kg (EBO-10), 25 mg/kg (EBO-25), or 100 mg/kg (EBO-100). Mean *Mab* burden in the lungs and standard error are shown (per group per time point, *n* = 5 at weeks −1, 0, and +1, and *n* = 10 at week +4).

ATCC 19977, M9501, and M9530 exhibit distinct susceptibility/resistance profiles to antibiotics commonly used to treat *Mab* disease (17), but EBO has the same MIC, 0.0625 µg/mL, against these isolates (Table 1) . To generate additional insight into the susceptibility of *Mab* to EBO *in vivo,* in a separate experiment, we assessed the efficacies of once daily 0.5, 5, 10, 25, and 100 mg/kg against *Mab* M9501, a recent isolate from a patient with lung disease, in the mouse model using the same protocol (Fig. 3). In mice that received 1× PBS, M9501 lung burden increased by 1.3 log_10_ CFU during the 4 weeks of treatment administration. Imipenem reduced M9501 lung burden by 2.2 log_10_ CFU at the end of 4 weeks of treatment. In mice that received 0.5 mg/kg EBO, lung M9501 burden increased throughout treatment duration albeit at a rate lower than in the mice that received PBS only. In mice that received 5 mg/kg or 10 mg/kg, reduction in lung M9501 burden at the completion of 1 and 4 weeks of treatment was almost identical. Both doses of EBO produced a net reduction of 0.4 log_10_ CFU at the completion of 4 weeks of treatment. In mice that received 25 mg/kg EBO, lung burden of M9501 decreased by 2.1 log_10_ CFU at the end of 4 weeks of treatment. In this assessment, the efficacy of 25 mg/kg EBO was similar to that of imipenem at the end of 4 weeks. In mice that received 100 mg/kg EBO, lung burden of M9501 decreased by 0.7 log_10_ CFU at 1 week and an additional 2.0 log_10_ CFU at the end of 4 weeks of treatment. With a net reduction of 2.7 log_10_ CFU, once-daily oral administration of 100 mg/kg EBO produced a larger reduction in M9501 burden than did twice-daily subcutaneous injection of 100 mg/kg imipenem. This difference could not be accounted for by any random variance within the two groups and therefore is significant (*P* = 0.007) (Table S3).

## DISCUSSION

The need for new antibiotics to improve treatment outcomes for patients with *Mab* lung disease is apparent as the microbiological response with existing treatments is estimated at less than 50% (24). Regimens that are included in the current recommendations to treat *Mab* disease (3, 4) include agents that inhibit cell-wall peptidoglycan synthesis (imipenem and cefoxitin), bacterial 50S ribosome function (clarithromycin, azithromycin, and linezolid), bacterial 30S ribosome function (amikacin, tigecyline), and yet-to-be-confirmed target in *Mab* (clofazimine) (25). Among the newer drugs that have demonstrated efficacy against *Mab* in laboratory studies and in the clinic, omadacycline inhibits protein synthesis (26), rifabutin inhibits RNA polymerase activity (27), and bedaquiline inhibits ATP synthase activity (28). Among the antibiotics that inhibit bacterial protein synthesis, EBO’s mechanism of action is novel, as it inhibits leucyl-tRNA synthetase activity (7, 9). As *Mab* is intrinsically resistant to most antibiotics, a new clinical candidate such as EBO that acts by inhibiting a unique target represents an opportunity to develop much-needed new therapeutics against this indication.

For EBO efficacy assessments, three *Mab* isolates with distinct MIC profiles against antibiotics frequently used to treat *Mab* disease were included. ATCC 19977 was included, as this strain is commonly used in laboratory studies of *Mab* and therefore can serve as the common denominator to compare findings of efficacies of different drugs and *Mab* isolates from independent studies (21). The criteria for selecting two additional *Mab* isolates were as follows: (1) they needed to be isolated recently from patients with lung infection in comparison to ATCC 19977, which was isolated in 1950 from a knee abscess, (2) one needed to be susceptible to most drugs included in the current *Mab* disease treatment guidelines, and (3) the other needed to be resistant to macrolides, one of the most commonly used classes of antibiotics used to treat *Mab* disease that, like EBO, is a protein synthesis inhibitor. M9501 was included as it met the first and second criteria; it is a recent clinical isolate that is susceptible to most of the drugs in the current roster to treat *Mab* disease (17). M9530 met the first and third criteria; the MIC of clarithromycin against this recent isolate is 3 µg/mL, which is classified as intermediate resistance according to CLSI breakpoints (17, 20).

Imipenem was included as a comparator for two reasons. Our foremost criterion was that imipenem is a standard-of-care agent. Second, imipenem exhibits bactericidal activity from the onset of treatment initiation. While the inclusion of more than one standard-of-care comparator would have yielded additional useful information, imipenem was chosen as the comparator as the primary goal of the efficacy study was to assess efficacy against one of the most active *Mab* drugs. We chose to administer imipenem at a dose of 100 mg/kg twice daily as previous studies have demonstrated that this regimen results in a rapid reduction in *Mab* burden in mouse lungs, making it a reliable comparator with high efficacy. However, it remains uncertain whether this dosage accurately reflects imipenem exposure in humans. Because mice and humans metabolize imipenem at different rates, primarily because of variations in dehydroxypeptidase-I activity (29), achieving equivalent exposure levels in mice that mimic those in humans is challenging. In humans, imipenem is often administered with cilastatin to protect it from dehydropeptidase-I and extend its half-life. However, since the equivalent dose of cilastatin in C3HeB/FeJ mice is unknown, cilastatin was not included in this study. Therefore, imipenem is included in this study not to accurately reflect the clinical dose in humans but rather to serve as a point of comparison.

In the studies shown in Fig. 2B and 3, there is a 0.9 log_10_ difference in the lung burden of M9501 at the conclusion. Both studies used the same protocol, mouse strain, and *Mab* isolate but could not be conducted simultaneously due to the large number of mice required for each study. Variations in the final M9501 lung burdens likely arose from biological differences between the cohorts, as it is challenging to exactly match the age and physiology of large numbers of mice, even when acquired from the same vendor. Another limitation of the study is that treatment was initiated at the conclusion of only 1 week of infection with *Mab*. Therefore, this stage of *Mab* infection is unlikely to represent the chronic disease seen in humans, which takes longer to develop. This limitation in the mouse model could be addressed by extending the *Mab* infection period to allow for the development of lung pathology that mimics chronic human disease before starting treatment.

These findings may have important clinical implications for the treatment of *Mab* lung disease in humans (3, 4). If once-daily oral 50 mg/kg EBO proves as effective against imipenem-resistant isolates such as M9530 or if once-daily oral 100 mg/kg that is more effective against clinical isolates such as M9501 in humans compared with twice-daily imipenem injections, EBO could become a valuable agent to treat *Mab* disease. Future studies on EBO’s efficacy against *Mab* lung disease, especially in combination with standard therapy, will be critical to determine its role in *Mab* treatment paradigm.

## MATERIALS AND METHODS

### Bacterial strains, growth media, drugs, and *in vitro* growth conditions

*Mab* isolates M9501, M9503, M9507, M9529, M9530, M9502, M9509, and M9514 were isolated between 2005 and 2015 from bronchiectasis or cystic fibrosis patients and obtained from Clinical Mycobacteriology Laboratory, Johns Hopkins University Hospital (30). The colony morphotype (smooth, rough, or mixed) is a notable and reversible characteristic of *Mab* isolates, except in specific genotypes such as 390R and 390S and depending on growth media (31). Due to the lack of widely accepted methods for determining colony morphotype, this assessment was not included in this study. Whole genome sequencing was undertaken in a prior study to determine subspecies of these isolates; M9501, M9503, M9507, M9529, and M9530 belong to the *abscessus* subspecies, and M9502, M9509, and M9514 belong to the *massiliense* subspecies (22). *Mab* strain ATCC 19977 and *Mycobacterium peregrinum* ATCC 700686 were purchased from American Tissue Type Collection (Manassas, VA). For the determination of MIC in accordance with the CLSI guidelines, all isolates were grown in Cation Adjusted Mueller-Hinton Broth (CAMHB) (Sigma-Aldrich, catalog # 90922) (20). For infecting mice, all *Mab* isolates were grown in Middlebrook 7H9 broth (Difco, catalog # 271310) supplemented with 0.5% glycerol, 10% albumin-dextrose-sodium chloride enrichment, and 0.05% Tween-80 as described (32) at 37°C in an orbital shaker at 220 RPM. Phosphate-buffered saline (PBS), pH 7.4, was purchased from Quality Biological (catalog # 114–058-101). EBO was provided by AN2 Therapeutics, imipenem was purchased from Octagon Chemicals Limited, and dexamethasone was purchased from Sigma-Aldrich (catalog # D1756). Middlebrook 7H11 selective agar (Difco, catalog # 283810) supplemented with 0.5% glycerol, 10% albumin-dextrose-sodium chloride enrichment, 50 µg/mL cycloheximide (Sigma-Aldrich, catalog # C7698), and 50 µg/mL carbenicillin (Research Products International, catalog # C46000) were used to culture *Mab* from mouse lung homogenates as described (32).

### Determination of MICs

MIC of EBO was determined using the standard broth microdilution method (33) with conditions specified for *Mab* and *M. peregrinum* in the CLSI guidelines (20). EBO was dissolved in sterile deionized water and filtered through a 0.22-µm filter to prepare stocks, and subsequently diluted to 1 mg/mL in CAMHB for use in MIC determination. Two-fold serial dilution was prepared to generate EBO concentration ranging from 0.0039 μg/mL to 2 μg/mL in 200 µL final volume per well of a 96-well microtiter culture plate. Using cultures of each isolate grown to exponential phase, 10^5^ CFU was inoculated into each well. Two wells containing 10^5^ CFU of each isolate and two wells containing CAMHB broth only were included in each plate as positive and negative controls for growth, respectively, and incubated at 30°C for 72 h as recommended by CLSI guidelines (20). Growth or lack thereof of *Mab* or *M. peregrinum* was assessed using Sensititre Manual Viewbox, and bacterial pellet size was recorded by normalizing to the size of the positive controls in the same plate. The lowest concentration of EBO at which a well suspension appeared identical to the well with broth alone was recorded as the MIC of EBO against the isolate. For each MIC determination, two biological duplicates with freshly grown cultures and two technical duplicates within each assay were performed, and the final MIC was calculated as the average of the biological and technical duplicates (Table 1; Table S1).

### Pharmacokinetic analysis

EBO pharmacokinetics were assessed in female C3HeB/FeJ mice by Quintara Discovery Inc. (Hayward, CA 94545). Epetraborole formulation and bioanalysis were performed as described (9). Thirty C3HeB/FeJ female mice, 5–6 weeks old, were dosed via oral gavage (PO) of 10, 50, 100, 200, and 300 mg/kg EBO solutions prepared in water adjusted to pH 5.02 with NaOH, and plasma concentrations of epetraborole were measured at the following time points 0.5, 2, 4, 8, 12, and 24 h post-dosing.

### Efficacy assessment of EBO in mice

Efficacy of EBO was undertaken as described in a previously published mouse model of lung *Mab* infection (14). In this model, all mice in an experimental cohort are placed in a chamber where they move freely. Infection results from the natural breathing of aerosolized *Mab* suspensions to mimic the natural route by which humans become infected with *Mab. Mab* burden in the lungs of mice at the end of 1, 2, and 4 weeks of treatment was considered end points to assess the efficacy of EBO against *Mab*. As comparators of treatment efficacy, both positive and negative control groups were included. In the positive control comparator group, mice received 100 mg/kg imipenem per dose, two doses daily, and in the negative control comparator group, mice were administered 1× PBS, the solvent used to deliver both EBO and imipenem.

C3HeB/FeJ mice (5–6 weeks old, female) were procured from Jackson Laboratories (Bar Harbor, ME) and allowed to acclimatize in the holding vivarium for 1 week prior to initiating studies. Beginning 1 week prior to infection and continuing through the study duration, 0.1 mL bolus of 1.25 mg/mL dexamethasone prepared in 1× PBS was administered once daily via subcutaneous injection. This dose is equivalent to 5 mg/kg/d. *Mab* isolates grown to the exponential phase were used to prepare a 10 mL suspension at A_600nm_ of 0.1 in Middlebrook 7H9 broth. Mice were placed in a Glas-Col chamber and exposed to aerosol generated from the *Mab* suspension according to the manufacturer’s instructions (Glas-Col, Terre Haute, Indiana). The efficacy of once-daily oral dosing of 25 mg/kg and 50 mg/kg EBO was assessed against three distinct *Mab* isolates: ATCC 19977, M9501, and M9530. Infection with each isolate was undertaken separately, and 90 mice were included in each infection. EBO doses of 25 mg/kg and 50 mg/kg were chosen so that the area-under-the-curve (AUC) exposures in mice were similar to the exposures achieved in humans with oral doses of 250 and 500 mg, respectively.

For each infection, five mice were sacrificed 24 h postinfection (designated week −1), lungs were homogenized in 1× PBS, appropriate dilutions were inoculated onto Middlebrook 7H11 agar, incubated at 37°C for 5 days, and CFU was enumerated to determine the *Mab* implanted at the time of infection. *Mab* infection was allowed to progress for 1 week prior to administering treatment to establish a robust infection and better model *Mab* lung disease state in humans. At 1-week postinfection (week 0), five mice were sacrificed, and *Mab* CFU was enumerated to determine the burden in the lungs at the initiation of treatment. On this day, mice were randomly allocated to four different groups, *n* = 20 per group. To each mouse in the first group, 0.2 mL bolus of sterile 1× PBS, pH 7.4, was administered once daily via oral gavage. To each mouse in the second group, 0.2 mL bolus of 12.5 mg/mL imipenem solution was administered twice daily via subcutaneous injection into the dorsal flank using a 27-gauge needle (equivalent to 100 mg/kg per dose, twice daily). To each mouse in the third group, 0.2 mL bolus of 3.125 mg/mL EBO solution was administered once daily via oral gavage (equivalent to 25 mg/kg, once daily). To each mouse in the fourth group, 0.2 mL bolus of 6.25 mg/mL EBO solution was administered once daily via oral gavage (equivalent to 50 mg/kg, once daily). EBO and imipenem solutions were prepared in sterile 1× PBS, pH 7.4. 1× PBS was used to dissolve both imipenem and EBO for administration to mice, unlike in the PK assessment where EBO was dissolved in water. Using a common solvent for both drugs tested in efficacy studies eliminated confounding variables associated with using different solvents. Daily refers to 7 days a week.

*Mab* burden in the lungs of mice was determined at the completion of 1, 2, and 4 weeks of treatment. To determine lung *Mab* burden in each treatment group, five mice per group were sacrificed at the completion of 1 and 2 weeks of treatment, and 10 mice per group were sacrificed at the completion of 4 weeks of treatment. *Mab* in the lung homogenates were cultured as described above. As the final time point is more informative about the long-term outcome of a treatment, a larger sample size was allocated to enable statistical assessment to distinguish smaller differences in *Mab* burden with higher certainty.

To generate additional insight into the potency of EBO against *Mab*, we determined the efficacy of once-daily oral administration of 0.5, 5, 10, 25, and 100 mg/kg EBO against *Mab* isolate M9501 in this mouse model. This study was undertaken with an identical protocol except for the end point at the completion of 2 weeks of treatment was excluded. One hundred fifteen mice were simultaneously infected with M9501. For the seven treatment groups in this assessment, which included five EBO groups, imipenem, and PBS only, five and 10 mice per treatment group were allocated for lung *Mab* CFU determinations at the completion of 1 and 4 weeks of treatment, respectively.

### Data analysis

*Mab* CFU data from efficacy studies were analyzed to determine mean and standard error at each time point in each study. GraphPad Prism V8.4.3 was used to perform *t*-tests (two samples assuming unequal variances) to assess variance between each treatment group at each time point. For comparison between groups, lung *Mab* burden in all mice in each group was compared. One-tailed *t*-test [*P*(T <= t)] are shown in the Table S3. Significance was determined at 95% confidence intervals. *P* ≤ 0.05 was interpreted as significant, and *P* > 0.05 was interpreted as not significant (represented as “ns”).

## SUPPLEMENTAL MATERIAL

Supplemental material aac.00648-24-s0001.pdf Fig. S1; Tables S1 to S3.

ASM does not own the copyrights to Supplemental Material that may be linked to, or accessed through, an article. The authors have granted ASM a non-exclusive, world-wide license to publish the Supplemental Material files. Please contact the corresponding author directly for reuse.
