# Supplementary material for: Efficacy of epetraborole against Mycobacteroides abscessus in a mouse model of lung infection
Source: Antimicrob Agents Chemother. 2024 Jul 17;68(8):e00648-24. doi: 10.1128/aac.00648-24 (PMC11323969; doi:10.1128/aac.00648-24)
Supplement: Supplemental material — Fig. S1; Tables S1 to S3. [file aac.00648-24-s0001.pdf]

SUPPLEMENTAL INFORMATION

Efficacy of epetraborole against *M. abscessus* in a mouse model of lung disease

Binayak Rimal[a], Christopher K. Lippincott[a,b], Chandra M. Panthi[a], Yi Xie[a], Tiffany R. Keepers[c], MRK Alley[c], Gyanu Lamichhane[a,b]

[a]Division of Infectious Diseases, Department of Medicine, School of Medicine, Johns Hopkins University, Baltimore, MD 21287, USA. [b]Center for Nontuberculous Mycobacteria and Bronchiectasis, School of Medicine, Johns Hopkins University, Baltimore, MD 21287, USA. [c]AN2 Therapeutics, Inc. Menlo Park, CA 94027, USA.

SUPPLEMENTARY FIGURES AND TABLES

```
Consensus   VTETQHDATDTAELPRHRYTAQLAGQIERRWQQTWADRGTFHVPNPVGSLAPTDGTPIPADKMFVQDMFPYPSGDGLHVGHPLGYIATDVYARFHRMRGANVLHALGFDAFGLPAEQYAV  120
19977_LeuS  VTETQHDATDTAELPRHRYTAQLAGQIERRWQQTWADRGTFHVPNPVGSLAPTDGTPIPADKMFVQDMFPYPSGDGLHVGHPLGYIATDVYARFHRMRGANVLHALGFDAFGLPAEQYAV  120
M9507_leuS  VTETQHDATDTAELPRHRYTAQLAGQIERRWQQTWADRGTFHVPNPVGSLAPTDGTPIPADKMFVQDMFPYPSGDGLHVGHPLGYIATDVYARFHRMRGANVLHALGFDAFGLPAEQYAV  120
M9529_LeuS  VTETQHDATDTAELPRHRYTAQLAGQIERRWQQTWADRGTFHVPNPVGSLAPTDGTPIPADKMFVQDMFPYPSGDGLHVGHPLGYIATDVYARFHRMRGANVLHALGFDAFGLPAEQYAV  120

Consensus   QTGTHPRVRTEANIVNYKRQLGRLGLGHDSRRSFATTDVDFYKWTQWIFLQIYNAWYDEQARRARPIQELIAEFDSGTRAPSDGTVWAELSVGARADVIDSYRLVYQSDSVVNWCPGLGT  240
19977_LeuS  QTGTHPRVRTEANIVNYKRQLGRLGLGHDSRRSFATTDVDFYKWTQWIFLQIYNAWYDEQARRARPIQELIAEFDSGTRAPSDGTVWAELSVGARADVIDSYRLVYQSDSVVNWCPGLGT  240
M9507_leuS  QTGTHPRVRTEANIVNYKRQLGRLGLGHDSRRSFATTDVDFYKWTQWIFLQIYNAWYDEQARRARPIQELIAEFDSGTRAPSDGTVWAELSVGARADVIDSYRLVYQSDSVVNWCPGLGT  240
M9529_LeuS  QTGTHPRVRTEANIVNYKRQLGRLGLGHDSRRSFATTDVDFYKWTQWIFLQIYNAWYDEQARRARPIQELIAEFDSGTRAPSDGTVWAELSVGARADVIDSYRLVYQSDSVVNWCPGLGT  240

Consensus   VLANEEVTADGRSERGNFPVFRKRLRQWMMRITAYSDRLIDDLDVLDWPDKVKTMQRNWIGRSQGASVLFGAPGVGDIEVFTTRPDTLFGATYMVLAPEHPLVDQLAADVWPQDADPRWT  360
19977_LeuS  VLANEEVTADGRSERGNFPVFRKRLRQWMMRITAYSDRLIDDLDVLDWPDKVKTMQRNWIGRSQGASVLFGAPGAGDIEVFTTRPDTLFGATYMVLAPEHPLVDQLAADVWPQDTDPRWT  360
M9507_leuS  VLANEEVTADGRSERGNFPVFRKRLRQWMMRITAYSDRLIDDLDVLDWPDKVKTMQRNWIGRSQGASVLFGAPGVGDIEVFTTRPDTLFGATYMVLAPEHPLVDQLAADVWPQDADPRWT  360
M9529_LeuS  VLANEEVTADGRSERGNFPVFRKRLRQWMMRITAYSDRLIDDLDVLDWPDKVKTMQRNWIGRSQGASVLFGAPGVGDIEVFTTRPDTLFGATYMVLAPEHPLVDQLAADVWPQDADPRWT  360

Consensus   GGQDSPRAAIEQYRRSIAAKSDLERQENKEKTGVFTGAYATNPVSGKPVPVFIADYVLLGYGTGAIMAVPGHDQRDWDFANTFGLPVQEVISGGDVTKAAYTGDGVLVNSDYLDGLDIEA  480
19977_LeuS  GGQDSPRAAIEQYRRSIAAKSDLERQENKEKTGVFTGAYATNPVSGKPVPVFIADYVLLGYGTGAIMAVPGHDQRDWDFANTFGLPVQEVISGGDVTKAAYTGDGVLVNSDYLDGLDIEA  480
M9507_leuS  GGQDSPRAAIEQYRRSIAAKSDLERQENKEKTGVFTGAYATNPVSGKPVPVFIADYVLLGYGTGAIMAVPGHDQRDWDFANTFGLPVQEVISGGDVTKAAYTGDGVLVNSDYLDGLDIEA  480
M9529_LeuS  GGQDSPRAAIEQYRRSIAAKSDLERQENKEKTGVFTGAYATNPVSGKPVPVFIADYVLLGYGTGAIMAVPGHDQRDWDFANTFGLPVQEVISGGDVTKAAYTGDGVLVNSDYLDGLDIEA  480

Consensus   AKVEVTRRLVKDGRGESRIEYKLRDWLFARQRYWGEPFPIVYDEDGRPRALGENVLPVELPEVEDYAPVSFDPDDASSEPSPPLSKAADWVNVELDLGDGLKHYTRDTNVMPQWAGSSWY  600
19977_LeuS  AKVEVTRRLVKDGRGESRIEYKLRDWLFARQRYWGEPFPIVYDEDGRPRALGENVLPVELPEVEDYAPVSFDPDDASSEPSPPLSKAADWVNVELDLGDGLKHYTRDTNVMPQWAGSSWY  600
M9507_leuS  AKVEVTRRLVKDGRGESRIEYKLRDWLFARQRYWGEPFPIVYDEDGRPRALGENVLPVELPEVEDYAPVSFDPDDASSEPSPPLSKAADWVNVELDLGDGLKHYTRDTNVMPQWAGSSWY  600
M9529_LeuS  AKVEVTRRLVKDGRGESRIEYKLRDWLFARQRYWGEPFPIVYDEDGRPRALGENVLPVELPEVEDYAPVSFDPDDASSEPSPPLSKAADWVNVELDLGDGLKHYTRDTNVMPQWAGSSWY  600

Consensus   ELRYADPDNAEAFCDKENEAYWLGPRPAEHGPNDPGGVDLYVGGMEHAVLHLLYSRFWHKVLYDLGHVSSREPYRRLLNQGYIQAHAYTDARGMYVPAAEVTEENGKFFYQGAEVQQEFG  720
19977_LeuS  ELRYADPDNAEAFCDKENEAYWLGPRPAEHGPNDPGGVDLYVGGMEHAVLHLLYSRFWHKVLYDLGHVSSREPYRRLLNQGYIQAHAYTDARGMYVPAAEVTEENGKFFYQGAEVQQEFG  720
M9507_leuS  ELRYADPDNAEAFCDKENEAYWLGPRPAEHGPNDPGGVDLYVGGMEHAVLHLLYSRFWHKVLYDLGHVSSREPYRRLLNQGYIQAHAYTDARGMYVPAAEVTEENGKFFYQGAEVQQEFG  720
M9529_LeuS  ELRYADPDNAEAFCDKENEAYWLGPRPAEHGPNDPGGVDLYVGGMEHAVLHLLYSRFWHKVLYDLGHVSSREPYRRLLNQGYIQAHAYTDARGMYVPAAEVTEENGKFFYQGAEVQQEFG  720

Consensus   KIGKSLKNSISPDDICDNYGADTLRVYEMSMGPLELSRPWATKDVVGAHRFLQRAWRVVVDEETGKIRVTTDDLTSEDTLRALHKTIAGVTEDYAALRNNTAAAKLIEYTNHLTKDYPEG  840
19977_LeuS  KIGKSLKNSISPDDICDNYGADTLRVYEMSMGPLELSRPWATKDVVGAHRFLQRAWRVVVDEETGKIRVTTDDLTSEDTLRALHKTIAGVTEDYAALRNNTAAAKLIEYTNHLTKDYPEG  840
M9507_leuS  KIGKSLKNSISPDDICDNYGADTLRVYEMSMGPLELSRPWATKDVVGAHRFLQRAWRVVVDEETGKIRVTTDDLTSEDTLRALHKTIAGVTEDYAALRNNTAAAKLIEYTNHLTKDYPEG  840
M9529_LeuS  KIGKSLKNSISPDDICDNYGADTLRVYEMSMGPLELSRPWATKDVVGAHRFLQRAWRVVVDEETGKIRVTTDDLTSEDTLRALHKTIAGVTEDYAALRNNTAAAKLIEYTNHLTKDYPEG  840

Consensus   APRAAVEPLVLMLAPLAPHLAEELWSLLGRDDSLAHGPFFESDDRWLVADTVEYPIQVNGKVRGRITVAADAPKGDIEAAALTEEKVLEFLAGATPKKVIVVPGRMVNLVV           951
19977_LeuS  APRAAVEPLVLMLAPLAPHLAEELWSLLGRDDSLAHGPFFESDDRWLVADTVEYPIQVNGKVRGRITVAADAPKGDIEAAALTEEKVLEFLAGATPKKVIVVPGRMVNLVV           951
M9507_leuS  APRAAVEPLVLMLAPLAPHLAEELWSLLGRDDSLAHGPFFESDDRWLVADTVEYPIQVNGKVRGRITVAADAPKGDIEAAALTEEKVLEFLAGATPKKVIVVPGRMVNLVV           951
M9529_LeuS  APRAAVEPLVLMLAPLAPHLAEELWSLLGRDDSLAHGPFFESDDRWLVADTVEYPIQVNGKVRGRITVAADAPKGDIEAAALTEEKVLEFLAGATPKKVIVVPGRMVNLVV           951
```

Figure S1: Alignment of LeuS sequences in *M. abscessus* reference strain ATCC 19977, and clinical isolates M9507 and M9529. Compared to LeuS in ATCC 19977, LeuS in M9507 and M9529 harbor A315V and T355A substitutions.

Table S1: MIC of epetraborole (EBO) against nine *M. abscessus* and a *M. peregrinum* isolate.

Isolate ID	Subspecies	Biological Replicate #1 MIC (μg/ml)		Mean MIC (μg/ml)	Biological Replicate #2 MIC (μg/ml)		Mean MIC (μg/ml)	Overall Mean MIC (μg/ml)
		Technical Replicate #1	Technical Replicate #2		Technical Replicate #1	Technical Replicate #2		
ATCC 19977	*abscessus*	0.0625	0.0625	0.0625	0.0625	0.0625	0.0625	0.0625
M9501	*abscessus*	0.0625	0.0625	0.0625	0.0625	0.0625	0.0625	0.0625
M9503	*abscessus*	0.0625	0.0625	0.0625	0.0625	0.0625	0.0625	0.0625
M9507	*abscessus*	>2	>2	>2	>2	>2	>2	>2
M9529	*abscessus*	1	1	1	1	1	1	1
M9530	*abscessus*	0.0625	0.0625	0.0625	0.0625	0.0625	0.0625	0.0625
M9502	*massiliense*	0.0625	0.0625	0.0625	0.0625	0.0625	0.0625	0.0625
M9509	*massiliense*	0.0313	0.0313	0.0313	0.0313	0.0313	0.0313	0.0313
M9514	*massiliense*	0.0625	0.0625	0.0625	0.0625	0.0625	0.0625	0.0625
700686	*peregrinum*	0.125	0.125	0.125	0.125	0.125	0.125	0.125

Minimum inhibitory concentration (MIC) of EBO was determined against six *M. abscessus* subspecies *abscessus,* and three *M. abscessus* subspecies *massiliense. Mycobacterium peregrinum* ATCC 700686 was included as an internal control comparator. MIC of EBO from two biological repeats and two technical repeats are shown. The final column shows the overall mean MIC calculated from the four MIC determinations shown in columns three, four, six and seven.

Table S2: Mean *M. abscessus* CFU burden in the lungs of mice in different treatment groups.

M. abscessus isolate	Figure panel in manuscript	Treatment Group	Mean *M. abscessus* CFU burden in the lungs (Log_{10})		
			Week +1	Week +2	Week +4
ATCC 19977	1A	PBS	5.11	4.90	5.25
		IMI	3.05	2.73	2.13
		EBO 25	3.66	3.86	2.97
		EBO 50	3.88	3.44	2.93
M9501	1B	PBS	4.71	5.47	6.68
		IMI	3.18	2.68	1.63
		EBO 25	4.15	4.16	2.71
		EBO 50	3.98	3.52	2.92
M9530	1C	PBS	4.92	5.37	7.18
		IMI	3.55	2.64	2.00
		EBO 25	3.92	3.62	3.47
		EBO 50	4.19	3.49	2.20
M9501	2	PBS	4.95	n/a	5.84
		IMI	3.68	n/a	2.34
		EBO 0.5	4.90	n/a	5.40
		EBO 5	4.81	n/a	4.14
		EBO 10	4.72	n/a	4.01
		EBO 25	4.31	n/a	2.42
		EBO 100	3.81	n/a	1.83

Mean burden of *M. abscessus* isolates ATCC 19977, M9501 and M9530 in the lungs of mice at the conclusion of one-, two- and four-weeks of treatment (represent as week +1, +2 and +4) are shown. *n*=5 mice per treatment group at week +1 and +2, and *n*=10 mice per treatment group at week +4. PBS: 1x phosphate-buffered-saline, pH 7.4. IMI: imipenem, 100 mg/kg*dose, dosed twice daily. EBO 0.5: epetraborole, 0.5 mg/kg, once daily. EBO 5: epetraborole, 5 mg/kg, once daily. EBO 10: epetraborole, 10 mg/kg, once daily. EBO 25: epetraborole, 25 mg/kg, once daily. EBO 50: epetraborole, 50 mg/kg, once daily. EBO 100: epetraborole, 100 mg/kg, once daily. n/a: not applicable as week +2 timepoint was not included for this specific assessment.

Table S3: Statistical assessment of *M. abscessus* burden in the lungs of mice between groups of mice receiving different treatments.

M. abscessus isolate	Treatment Group	*p*-value from *t*-Test: Two-Sample Assuming Unequal Variances (P(T<=t) one-tail)					
		Week +1	*p*-value interpretation	Week +2	*p*-value interpretation	Week +4	*p*-value interpretation
ATCC 19977	PBS vs. IMI	0.001	**	<0.001	**	<0.001	**
	PBS vs. EBO 25	0.004	**	0.019	*	0.001	**
	PBS vs. EBO 50	<0.001	**	<0.001	**	<0.001	**
	IMI vs. EBO 25	0.091	ns	0.017	*	0.018	*
	IMI vs. EBO 50	0.022	*	0.029	*	0.013	*
	EBO 25 vs. EBO 50	0.257	ns	0.163	ns	0.459	ns
M9501	PBS vs. IMI	0.031	*	<0.001	**	<0.001	**
	PBS vs. EBO 25	0.235	ns	0.015	*	<0.001	**
	PBS vs. EBO 50	0.157	ns	0.002	**	<0.001	**
	IMI vs. EBO 25	0.044	*	0.010	**	0.006	**
	IMI vs. EBO 50	0.035	*	0.070	ns	0.002	**
	EBO 25 vs. EBO 50	0.382	ns	0.108	ns	0.334	ns
M9530	PBS vs. IMI	0.016	*	0.001	**	<0.001	**
	PBS vs. EBO 25	0.055	ns	0.006	**	<0.001	**
	PBS vs. EBO 50	0.081	ns	0.004	**	<0.001	**
	IMI vs. EBO 25	0.229	ns	0.045	*	<0.001	**
	IMI vs. EBO 50	0.056	ns	0.058	ns	0.183	ns
	EBO 25 vs. EBO 50	0.266	ns	0.398	ns	<0.001	**
M9501	PBS vs. IMI	0.014	*	n/a	n/a	<0.001	**
	PBS vs. EBO 0.5	0.458	ns	n/a	n/a	0.228	ns
	PBS vs. EBO 5	0.396	ns	n/a	n/a	0.228	ns
	PBS vs. EBO 10	0.332	ns	n/a	n/a	0.001	**
	PBS vs. EBO 25	0.083	ns	n/a	n/a	<0.001	**
	PBS vs. EBO 100	0.018	*	n/a	n/a	<0.001	**
	IMI vs. EBO 0.5	0.011	*	n/a	n/a	<0.001	**
	IMI vs. EBO 5	0.015	*	n/a	n/a	<0.001	**
	IMI vs. EBO 10	0.020	*	n/a	n/a	<0.001	**
	IMI vs. EBO 25	0.025	*	n/a	n/a	0.346	ns
	IMI vs. EBO 100	0.325	ns	n/a	n/a	0.007	**
	EBO 0.5 vs. EBO 5	0.435	ns	n/a	n/a	0.011	*
	EBO 0.5 vs. EBO 10	0.366	ns	n/a	n/a	0.005	**
	EBO 0.5 vs. EBO 25	0.085	ns	n/a	n/a	<0.001	**
	EBO 0.5 vs.EBO 100	0.017	*	n/a	n/a	<0.001	**
	EBO 5 vs. EBO 10	0.430	ns	n/a	n/a	0.350	ns
	EBO 5 vs. EBO 25	0.115	ns	n/a	n/a	<0.001	**
	EBO 5 vs. EBO 100	0.023	*	n/a	n/a	<0.001	**
	EBO 10 vs. EBO 25	0.151	ns	n/a	n/a	<0.001	**
	EBO 10 vs. EBO 100	0.030	*	n/a	n/a	<0.001	**
	EBO 25 vs. EBO 100	0.026	*	n/a	n/a	0.008	**

Results of two tailed *t*-test of *M. abscessus* burdens in the lungs of mice in different treatment and infection groups at 1-, 2- and 4-week timepoints are shown. The mean *M. abscessus* lung burdens at 0-, 1-, 2- and 4-week timepoints following treatment are illustrated in Figures 1 and 2 of the manuscript. n=5 mice per treatment group at week +1 and +2, and n=10 mice per treatment group at week +4. Column 1 lists the infecting *M. abscessus* isolate. Column 2 lists the pairs of treatment groups within the study. PBS: 1x phosphate-buffered-saline, pH 7.4. IMI: imipenem, 100 mg/kg*dose, dosed twice daily. EBO 0.5: epetraborole, 0.5 mg/kg, once daily. EBO 5: epetraborole, 5 mg/kg, once daily. EBO 10: epetraborole, 10 mg/kg, once daily. EBO 25: epetraborole, 25 mg/kg, once daily. EBO 50: epetraborole, 50 mg/kg, once daily. EBO 100: epetraborole, 100 mg/kg, once daily. n/a: not applicable as week +2 timepoint was not included for this specific assessment. * represents p-value ≤0.05, and ** represents p-value of ≤0.01, and were interpreted as significant. "ns" represents a p-value >0.05 that was interpreted as not significant.
